# Giant rectus sheath hematoma

**DOI:** 10.1186/s13089-019-0129-4

**Published:** 2019-06-18

**Authors:** Gabriela Bello, Pablo Blanco

**Affiliations:** 1 Intensive Care Unit, Hospital Central de las Fuerzas Armadas (DNSFFAA), 3060, 8 de Octubre Ave., 11600 Montevideo, Uruguay; 2 Intensive Care Unit, Clínica Cruz Azul, 2651, 60 St., Necochea, 7630 Argentina

**Keywords:** Rectus abdominis, Hematoma, Point of care, Ultrasound, Computed tomography, Critical care

## Abstract

**Background:**

Rectus sheath hematoma (RSH) is an uncommon entity associated with predisposing factors such as anticoagulation. It may mimic more frequent abdominal conditions and its accurate diagnosis is important to focus on the correct treatments and improve morbidity and mortality.

**Case presentation:**

An elderly patient with shock, abdominal pain, palpable abdominal mass, and anemia was suspected of having a large RSH by point-of-care ultrasound (POCUS), which was then confirmed by computed tomography. Surgery was performed, markedly improving his clinical status.

**Conclusions:**

POCUS has a good sensitivity for the diagnosis of RSH and it is also an excellent tool for patient follow-up.

**Electronic supplementary material:**

The online version of this article (10.1186/s13089-019-0129-4) contains supplementary material, which is available to authorized users.

## Background

Rectus sheath hematoma (RSH) is an uncommon entity, accounting for less than 2% of patients complaining of acute abdominal pain [1]. It may mimic several more common conditions, such as intestinal obstruction, perforated peptic ulcer, pancreatitis, diverticulitis, tumors or a ruptured aortic aneurysm [2, 3]. Thus, a high index of suspicion and a careful diagnostic workup are mandatory to reach an accurate diagnosis, so as to focus on the correct treatment.

Known risk factors predisposing to RSH are anticoagulation (nearly invariably present); older age; female sex; pregnancy; trauma; iatrogenic/surgery; chronic medical conditions, such as hypertension, atherosclerosis or hematologic diseases; coughing and forceful rectus muscle contractions [2–4].

The overall mortality rate of RSH is around 4% and rises up to 25% in anticoagulated patients due to increased hemorrhage volume [2]; an early recognition of RSH may be associated with improved chances of survival [5].

We present the case of a patient with a large RSH suspected by the findings of point-of-care ultrasound (POCUS) and confirmed by computed tomography (CT) and discuss the importance of POCUS in the diagnosis and management of patients with this condition.

## Case presentation

**Fig. 1:**
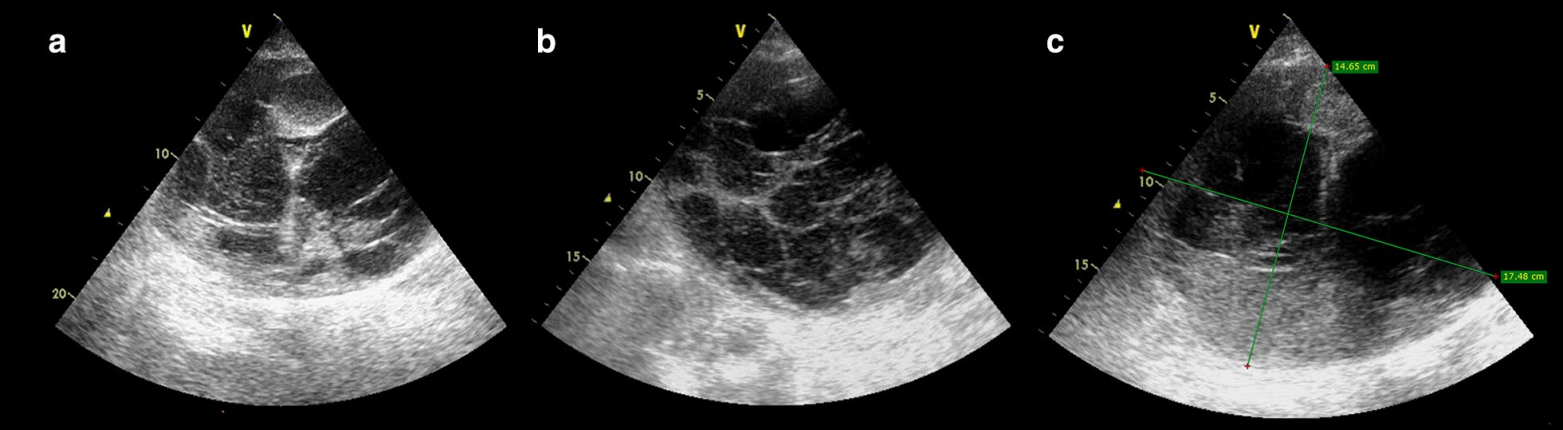
Abdominal ultrasound from the left coronal view showing a complex cystic mass (**a** and **b**) with multiple internal septations, measuring approximately 18 cm × 15 mm (**c**), highly suggestive of a large rectus sheath hematoma (RSH)

**Fig. 2:**
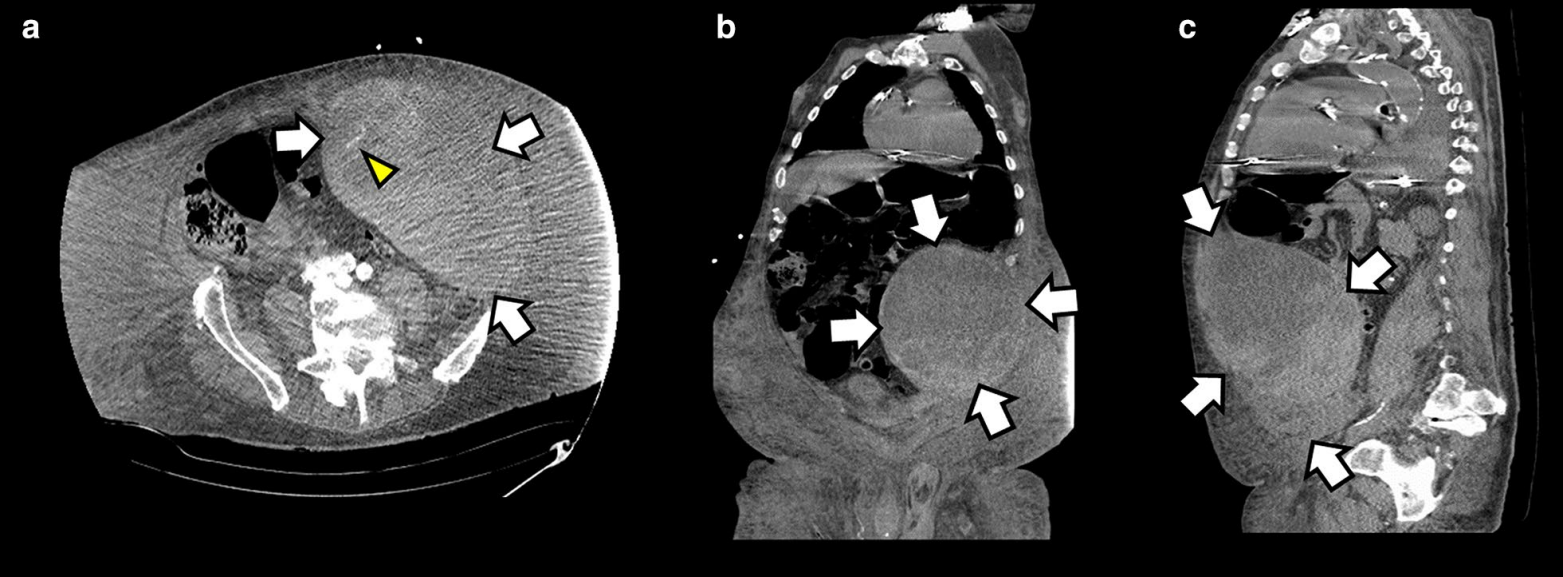
Abdominopelvic computed tomography with intravenous contrast showing a large left RSH (arrows) extending to the pelvis. Signs of active contrast extravasation are shown in **a** (arrowhead), indicating active bleeding; **a** axial plane; **b** coronal plane; **c** sagittal plane

An 85 year-old-male patient with a history of congestive heart failure and atrial fibrillation under anticoagulation with enoxaparin was admitted to the intensive care unit (ICU) presenting with shock and abdominal pain. Extremely pale skin and mucous membranes were noted, along with an extensive ecchymosis occupying the left hemiabdomen (i.e., Grey Turner´s sign). The abdominal mass bulged on the left flank and was soft, painful and non-pulsating on palpation. Blood chemistry highlighted anemia (hemoglobin 5.7 g/dl), metabolic acidosis and elevation of creatinine and BUN; coagulation tests were normal. A POCUS abdominal ultrasound of the left flank (Fig. 1 and Additional file 1: Video 1) showed a large complex avascular cystic mass which extended to the pelvis, with echogenic particulate mobile contents in dependent areas, and multiple internal septations. Perisplenic free peritoneal fluid was also noted. No other anomalies were found. Given the described ultrasound features and in the context of this clinical presentation together with anemia, this mass was interpreted as a hematoma. However, because of its size, it could not be concluded with certainty whether it was an extra-abdominal (e.g., abdominal wall) or an intraabdominal structure. Thus, an abdominopelvic CT with intravenous contrast was ordered. The CT showed a large heterogeneous fluid collection along the left rectus sheath which extended to the subperitoneal space (Fig. 2), showing signs of active contrast extravasation, suggestive of active bleeding (Fig. 2a). With the diagnosis of a large RSH complicated with hemodynamic instability, suspicion of abdominal compartment syndrome, and signs of active bleeding on the CT, the patient was immediately transferred to the operating room for surgical exploration. On laparotomy, 3 L of fresh blood mixed with clots was evacuated from the abdominal wall and subperitoneal space. The culprit vessel was identified as a branch of the left inferior epigastric artery, which was then successfully ligated. The patient´s hemodynamic parameters markedly improved after surgery and blood transfusions, and he was sent back to the ICU for further care, where he developed acute renal failure requiring hemodialysis. During the follow-up period, POCUS did not show signs of rebleeding within the rectus sheath. The patient died in the general ward after a prolonged length of stay in the ICU. The main complications observed were renal failure requiring hemodialysis as mentioned before, and nosocomial infections.

## Discussion

**Fig. 3:**
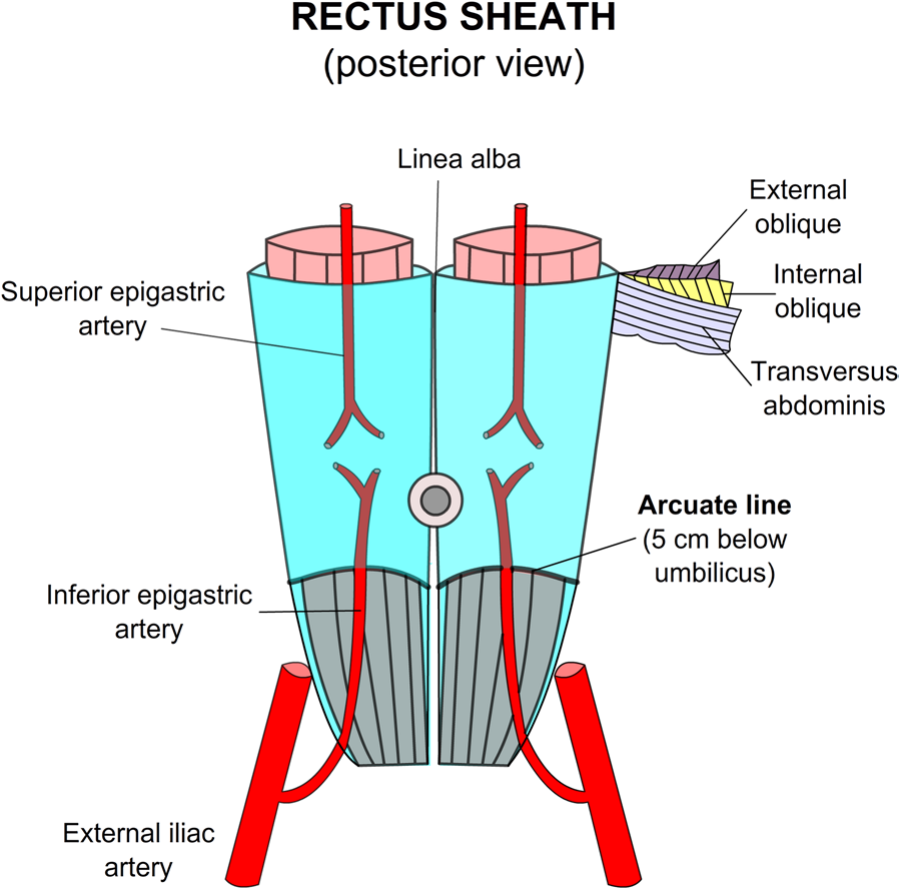
Anatomy of the rectus sheath above and below the arcuate line observed from a posterior view. As noted, below the arcuate line, the posterior sheath is absent and thus the rectus muscle is in direct contact with the transversalis fascia

Rectus sheath hematoma results from bleeding into the rectus sheath, after damage to the epigastric arteries or by direct muscle tear [2]. RSH is frequently localized below the umbilicus where the inferior epigastric artery (branch of the external iliac artery) penetrates the rectus muscles at the arcuate line and this vessel is relatively fixed, so branches are more prone to being injured [6]. Furthermore, below the arcuate line, which is approximately 5 cm below the umbilicus, all the aponeuroses pass in front of the rectus abdominal muscle, thus creating a possibility for the hematoma to spread between the rectus muscle and fascia transversalis into the prevesical space, without other supporting planes (Fig. 3). Bleeding from the superior epigastric artery (terminal branch of the internal thoracic artery) is less common and minor, given the small caliber of this vessel as well as the effective supporting mechanism of the posterior rectus sheath [7].

The most common presentation of RSH is abdominal pain and a palpable abdominal mass [3, 4], followed by anemia [4]; very few patients may present with hemodynamic instability [3].

To reach a diagnosis, the clinical clues orienting toward an RSH are Carnett’s sign (i.e., the abdominal pain worsens after voluntary contraction of the abdominal muscles), Fothergill´s sign (i.e., the mass remains palpable after voluntary contraction of the abdominal muscles) and Cullen’s (i.e., periumbilical ecchymosis) and Grey Turner’s (i.e., flank ecchymosis) signs. However, clinical data are fairly unreliable, with a failure rate higher than 50% for the diagnosis of an RSH [1]. Conversely, diagnosis of RSH relies upon the use of imaging techniques. Given its widespread availability, bedside use, non-invasiveness, repeatability, lack of ionizing radiation and low costs, POCUS should be considered the first-line method, both to diagnose RSH and to rule in or out other common etiologies (e.g., ruptured abdominal aneurysm). Sensitivity of ultrasound for an RSH is around 90%, which indicates that some patients still remain undiagnosed using this technique [1, 2]. In addition, particularly for large hematomas, determining the exact origin of the mass is nearly impossible, and thus a CT is often required in these settings [2]. For abdominopelvic CT, the reported sensitivity and specificity for the diagnosis of an RSH reaches 100% and thus this method is often performed when the ultrasound is unreliable or a large hematoma is observed and active bleeding is suspected, proving that demonstrating signs of active bleeding, in addition to hemodynamic instability, points toward a non-conservative (embolization or surgery) treatment [2, 7]. Based on CT, a classification for RSH is well described [8] and this can be readily extrapolated to ultrasound (Table 1). Also, CT may even rule in or out other common diagnoses which could be missed by ultrasound. Common problems with CT are the need to transfer the patient to the radiology department and the use of ionizing radiation as well as iodinated contrast agents, which expose patients to allergic reactions as well as contrast-induced nephrotoxicity (CIN). Weighing the risks of CIN versus leaving an RSH undiagnosed should be assessed on an individual basis. In these cases, performing a CT without contrast media is a valid option, but demonstrating signs of active bleeding as well as eventually reaching an accurate diagnosis of other common pathologies will not be possible [9].

**Table 1:** Rectus sheath hematoma classification based on computed tomography. Modified from [8]

Type I	The hematoma is intramuscular and an increase in muscle size is observed, with an ovoid or fusiform aspect and hyperdense foci or diffusely increased density. The hematoma is unilateral and does not dissect along adjacent fascial planes
Type II	The hematoma is intramuscular (mimicking type I), but with blood between the muscle and the transversalis fascia. It may be uni- or bilateral, and no blood is seen occupying the prevesical space
Type III	The hematoma may or may not involve the muscle, and blood is seen between the transversalis fascia and the muscle, in the peritoneum, and in the prevesical space

**Fig. 4:**
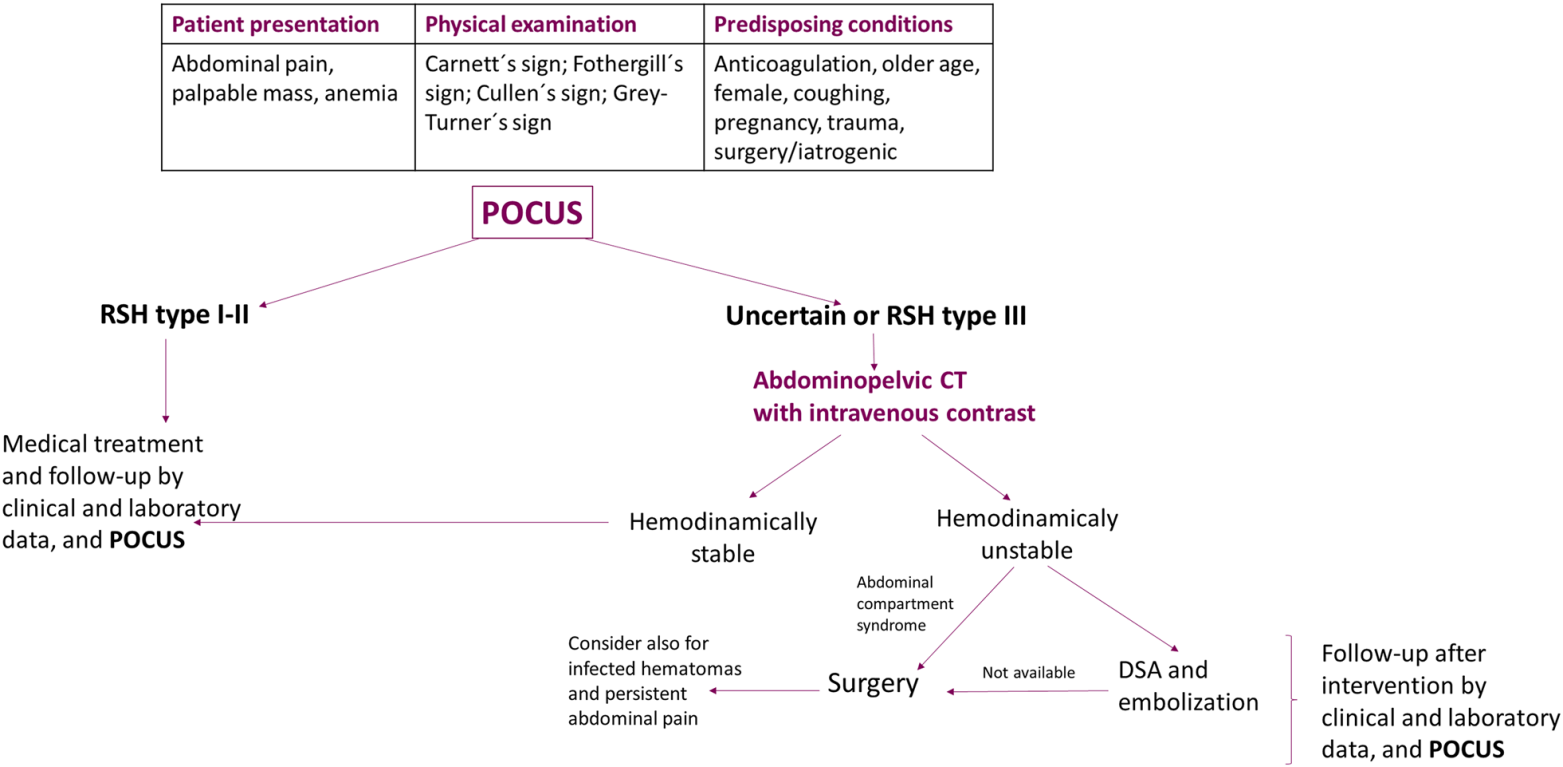
Algorithm for diagnosing and managing a patient with an RSH. Modified from [2, 5]; RSH, rectus sheath hematoma; POCUS, point-of-care ultrasound; CT, computed tomography; DSA, digital subtraction angiography

Conservative treatment is effective in the majority of patients with RSH. This group of patients is followed up by clinical and laboratory data, as well as serial ultrasound (US) examinations, measuring the size of the hematoma. US-guided drainage does not offer clear advantages compared to conservative treatment and may even prolong the length of hospital stay and predispose to a hematoma infection. Thus, this practice should not be routinely recommended [6]. However, the use of US-guided diagnostic aspiration may be considered in those patients managed conservatively in whom an infected hematoma is suspected. On the other hand, patients with hemodynamic instability, persistent abdominal pain, those with suspicion of infected hematomas, as well as patients with elevated intraabdominal pressures developing an abdominal compartment syndrome (ACS) often need a more aggressive approach [2, 4, 7, 9]. Digital subtraction angiography (DSA) and selective embolization of the culprit vessel represent the preferred approach for unstable patients, with a high degree of success and low rebleeding risk [10, 11]. Surgery is indicated in cases when embolization is not available, the patient evolves to an ACS or the hematoma is infected, allowing for the evacuation of the hematoma, lowering the intraabdominal pressure and performing the ligation of the culprit vessel [5]. For patients in whom embolization or surgery was performed, postoperative follow- up with serial US examinations may allow practitioners to detect early rebleeding signs within the rectus sheath as well as to identify possibly infected contents, for which US-guidance diagnostic aspiration may be useful. An algorithm for the diagnosis and management of the patient with RSH is provided in Fig. 4.

## Conclusions

RSH, although uncommon, should be considered in any patient presenting with abdominal pain. Abdominal POCUS, as the first-line imaging modality for the patient in the ICU or the emergency department, may allow practitioners to reach this diagnosis in most of the patients, avoiding the performance of unnecessary tests and the administration of wrong treatments, and allowing to selectively use more costly, sophisticated or potentially harmful methods, including the use of iodinated contrast media, in certain cases requiring so. POCUS should also be considered as a suitable method for the follow-up period.

## Additional file

**Additional file 1: Video 1.** Abdominal ultrasound from the left coronal view showing a large complex cystic mass with multiple internal septations, corresponding to a large rectus sheath hematoma.

## Abbreviations

RSH: rectus sheath hematoma
ICU: intensive care unit
POCUS: point-of-care ultrasound
CT: computed tomography
DSA: digital subtraction angiography
ACS: abdominal compartment syndrome
